# Primary Diffuse Large B-cell Lymphoma of the Sigmoid Colon

**DOI:** 10.7759/cureus.5048

**Published:** 2019-06-30

**Authors:** Ibrahim Haddad, Bara El Kurdi, Mahmoud El Iskandarani, Sumbal Babar, Mark Young

**Affiliations:** 1 Internal Medicine, Quillen College of Medicine, East Tennessee State University, Johnson City, USA; 2 Internal Medicine, East Tennesee State University, Johnson City, USA; 3 Internal Medicine, East Tennessee State University, Johnson City, USA

**Keywords:** primary gastrointestinal lymphoma, primary colorectal lymphoma, primary diffuse large b-cell lymphoma of the colon, large colon

## Abstract

Primary gastrointestinal lymphoma is the most common type of extra-nodal lymphoma, representing about 30%-50% of all extra-nodal involvement. The stomach is the most common site, with the colon and rectum accounting for a minority of occurrences. Primary colorectal lymphoma is uncommon, representing only 0.3% of all large intestinal malignancies and approximately 3% of gastrointestinal (GI) lymphomas, with the majority of these being B-cell non-Hodgkin lymphoma and diffuse large B-cell lymphoma (DLBCL) being the most common subtype. We present a case of an 85-year-old male who presented with symptoms suggestive of bowel obstruction, who, after further evaluation, was diagnosed with primary non-Hodgkin lymphoma of the colon, DLBCL subtype.

## Introduction

The gastrointestinal(GI) tract is the most common site of primary extra-nodal lymphoma, with the vast majority being non-Hodgkin lymphomas (NHL). The colon is the primary site in around 17% of cases. Diffuse large B-cell lymphoma (DLBCL) of the colon is the most common subtype of extranodal non-Hodgkin's lymphoma, and while there is conflicting data regarding the most common part of the colon being involved, most studies report the cecum as the most common location. Due to the rarity of reported cases, there is no agreeable protocol on how to manage primary DLBCL of the colon. Currently, surgical resection is the cornerstone of treatment followed by the cyclophosphamide, doxorubicin, vincristine, and prednisone (CHOP) regimen-based chemotherapy and/or radiation therapy. The role of targeted therapy and immunotherapy is still being evaluated but may be a promising alternative to the present standard treatment. We present a case of primary DLBCL of the colon in an 85-year-old male patient followed by a literature review and discussion of the reported cases in the literature.

## Case presentation

**Figure 1:**
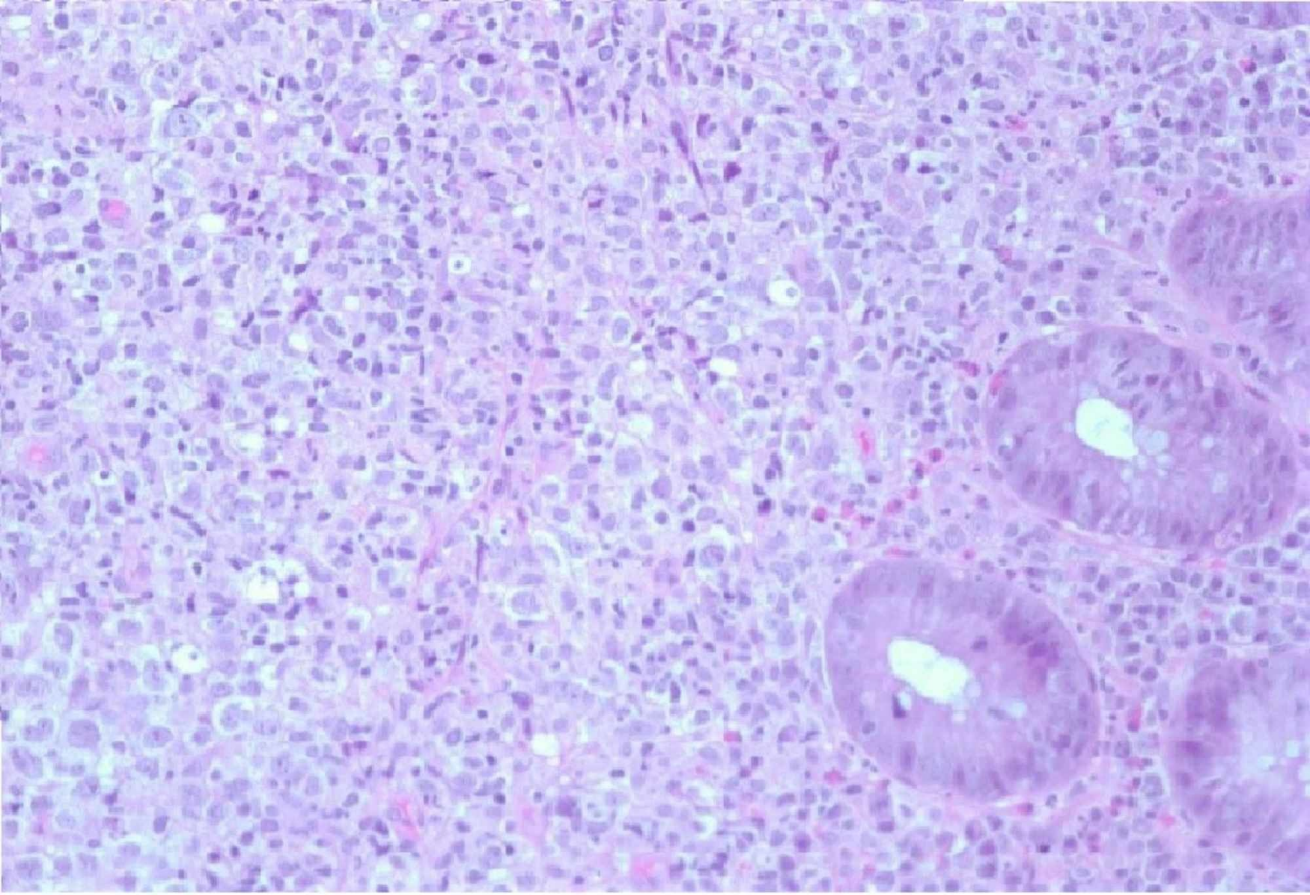
400 power microscope representing our patient's primary DLBCL of the colon

**Figure 2:**
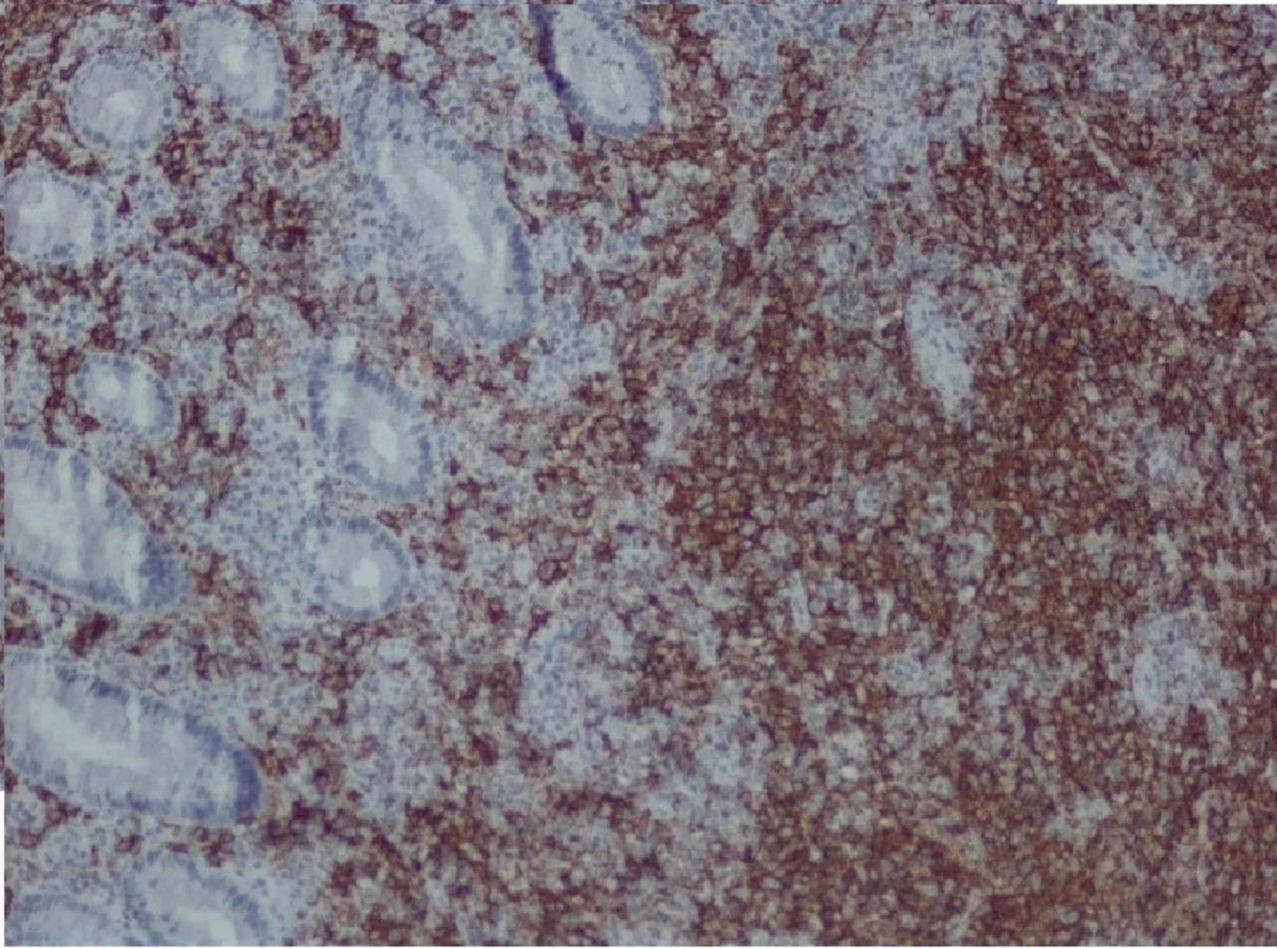
CD 20 stain for our patient's primary DLBCL of the colon

An 85-year-old male with a history of atrial fibrillation presented to the hospital with acute shortness of breath and was subsequently admitted under the diagnosis of acute heart failure exacerbation. At admission, the patient was found to be anemic. His last colonoscopy was five years ago, with findings of lipoma in the ascending colon as well as sigmoid diverticulosis. At the time of admission, he had been more confused per his daughter. He was complaining of progressive fatigue and struggling with constipation, as he had been without bowel movement for several days prior to admission. He also had decreased appetite and lost around six pounds in the past four months. The patient subsequently had a chest X-ray that demonstrated multiple rounded pulmonary nodules throughout the lungs. The patient was clinically diagnosed with bowel obstruction. He subsequently underwent a computed tomography (CT) scan of the abdomen and pelvis, which showed a long apple-core lesion seen in the mid to distal sigmoid colon, which was about six cm in length with well-defined extension into the mesentery and discrete retroperitoneal lymphadenopathy. The patient then underwent a colonoscopy, which showed a near-complete obstructing sigmoid mass with ulceration. Multiple biopsies were taken from this sigmoid mass. The pathological evaluation was positive for atypical lymphoid cells (Figure 1) that had convoluted nuclei with multiple prominent nuclei and displayed elevated mitotic activity. An immune panel revealed neoplastic cells that were positive for CD 20 (Figure 2), BCL 6, CD10, and CD 23 and were negative for CD3, CD5, M EOM -1, and cyclin D1k. The patient was diagnosed with primary DLBCL of the sigmoid colon and treatment options were discussed with him and his family. The patient refused treatment and instead chose to proceed with inpatient hospice care. Unfortunately, he subsequently passed away during this admission.

## Discussion

The gastrointestinal (GI) tract is the most common site of primary extra-nodal lymphoma, with the vast majority being non-Hodgkin lymphomas. In recent years, there has been an increase in the incidence of primary GI non-Hodgkin lymphoma. A retrograde study [1] done in Alberta, Canada, in 2008, determined the incidence of primary GI non-Hodgkin lymphoma over a 10-year period from January 1999 to January 2009, with a total study population of 1,285,972. The study identified a total of 149 cases of primary GI NHL during the study period. Age and sex-adjusted yearly incidence rates ranged from 0.13 per 100,000 in 1999 to 2.39 per 100,000 in 2007. Diffuse large B-cell lymphoma was the most common histological subtype. Specifically, the stomach was the most common site of GI involvement and comprised 47% of diffuse large B-cell lymphoma (DLBCL). The colon was the primary site in 17% of the cases. The study concluded that in all instances, with the exception of the esophagus, in which only one case in 10 years was found, the annual incidence rates of GI NHL according to the site increased over time.

A retrograde study [2] that was done in Mashhad, Iran, at the Radiation Oncology department of Omid Hospital assessed the clinical presentation of primary GI lymphoma as well. The study included a total number of 30 cases with primary GI lymphoma, all of them being identified as non-Hodgkin lymphoma, over a period of five years from 2006-2011. The clinical presentation of these cases with a mean age of patients was 50 ± 16.9 years (range: 15-79 years). The most common presenting symptoms were dyspepsia and obstruction. B symptoms were present in 27 patients (90%). Lactate dehydrogenase (LDH), an important prognostic factor in non-Hodgkin's lymphoma, was elevated in nine patients (32.1%). Anemia was present in 20 patients (66.6%). In most patients, other laboratory tests such as platelets, white blood cell count, and liver enzymes were normal.

The colon is a rare location for GI lymphoma, representing only 3% of all cases [3], with the cecum being the most common site [4] and the most common subtype of primary colorectal lymphoma being diffuse large B-cell lymphoma [5]. No specific risk factors have been identified for diffuse large B-cell lymphoma of the colon and rectum. However, the condition may be associated with autoimmune disorders, inflammatory bowel disease (IBD), advanced age, and immunodeficiency (human immunodeficiency virus (HIV) infection, organ transplant, etc.). Presenting symptoms can be varied, including abdominal pain, colonic obstruction, diarrhea, lower GI bleeding, fever, night sweats, weight loss, and, in rare cases, colon perforation. The diagnosis is often established after biopsy during colonoscopy or after pathological analysis of the colon post colectomy or hemicolectomy performed in cases of colonic perforation.

DLBCL staging involves a CT or magnetic resonance imaging (MRI) scan, positron emission tomography (CT-PET) scan, and/or bone marrow biopsy in the case of advanced stages to determine bone marrow involvement. If neurological symptoms are present and central nervous system involvement is suspected, brain imaging and lumbar puncture might also be considered. Recent advances in gene expression profiling and immunohistochemical (IHC) analysis on tissue biopsy have allowed us to differentiate the subtypes of DLBCL and tailor treatment to each type. For example, patients with the activated B-cell (ABC) disease subtype are less likely to respond well to cyclophosphamide, doxorubicin, vincristine, and prednisone (CHOP)-based regimens than those with germinal center B-cell (GCB) disease [6]. Cornerstone treatment options include chemotherapy, radiation therapy, surgery, bone marrow transplantation, or stem cell transplantation, with the latter two options implemented if response to treatment is not complete or if the chance of recurrence is high, with radiation therapy being the least preferred due to a high risk of small and large intestine complications [7]. Given the current era of target and immunotherapy, a growing amount of evidence indicates that both options are viable in the management of some cases. Multiple retrospective studies found that rituximab treatment, in addition to chemotherapy, may improve clinical outcomes [8-9]. Despite advancements in treatment options, primary DLBCL of the colon remains an aggressive disease with a poor prognosis.

 In our review of the literature, we found multiple reported cases and case series, however, we only cited those that were crucial for the purposes of this report. We will give a brief discussion regarding the presenting epidemiology, presentation, and treatment options of these cases. The male to female ratio of the reported cases was 2.2:1, with 13 male patients and six female patients. The mean age was 68 years old. From the 19 cases, 18 mentioned the location of the lymphoma as following: sigmoid 44%, cecum 39%, and the ascending colon 17%. Presenting symptoms include abdominal pain, weight loss, diarrhea, constipation, lower GI bleeding, vesicocolic fistula, and altered mental status. The most common presenting symptoms were bowel obstruction and abdominal pain, present in 40% of 15 cases. Three patients had colonic perforation during admission, representing about 16% of the patient population. Sixty-eight percent of the patients had colectomy or hemicolectomy as part of their treatment with CHOP as the main chemotherapeutic regimen based upon the International Prognostic Index (IPI). The IPI is a common prognostic scoring system used for DLCBL, and it is also useful for primary DLCBL of the colon [10].

## Conclusions

Primary DLCBL of the colon is a rare entity representing only 1% of all colon malignancy, with the cecum being the most common location. Diagnosis is often established after a pathological evaluation of colonic biopsy or after colonic resection. Advances in gene expression profiling and immunohistochemical analysis (IHC) have allowed for tailored treatment for each type. Patients with the ABC disease subtype are less likely to respond well to CHOP-based regimens than those with GCB disease. Primary DLBCL of the colon remains an aggressive disease with a poor prognosis. Treatment options include surgical resection, chemotherapy, and radiation therapy. A bone marrow transplant is considered in select cases. Given the current era of rapid advancements in technology, targeted therapy and immunotherapy should also be considered as potential therapeutic options.
